# Simulating school closure policies for cost effective pandemic decision making

**DOI:** 10.1186/1471-2458-12-449

**Published:** 2012-06-18

**Authors:** Ozgur M Araz, Paul Damien, David A Paltiel, Sean Burke, Bryce van de Geijn, Alison Galvani, Lauren Ancel Meyers

**Affiliations:** 1 College of Public Health, University of Nebraska Medical Center, Omaha, NE 68198, USA; 2 Information Risk and Operations Management Department, McCombs School of Business, The University of Texas, Austin, TX, 78712, USA; 3 School of Medicine, Yale University, CT, New Haven, 06510, USA; 4 Section of Integrative Biology, The University of Texas at Austin, Austin, TX, 78712, USA; 5 Epidemiology and Public Health, New Haven, CT, 06510, USA; 6 Santa Fe Institute, Santa Fe, NM, USA

## Abstract

**Background:**

Around the globe, school closures were used sporadically to mitigate the 2009 H1N1 influenza pandemic. However, such closures can detrimentally impact economic and social life.

**Methods:**

Here, we couple a decision analytic approach with a mathematical model of influenza transmission to estimate the impact of school closures in terms of epidemiological and cost effectiveness. Our method assumes that the transmissibility and the severity of the disease are uncertain, and evaluates several closure and reopening strategies that cover a range of thresholds in school-aged prevalence (SAP) and closure durations.

**Results:**

Assuming a willingness to pay per quality adjusted life-year (QALY) threshold equal to the US per capita GDP ($46,000), we found that the cost effectiveness of these strategies is highly dependent on the severity and on a willingness to pay per QALY. For severe pandemics, the preferred strategy couples the earliest closure trigger (0.5% SAP) with the longest duration closure (24 weeks) considered. For milder pandemics, the preferred strategies also involve the earliest closure trigger, but are shorter duration (12 weeks for low transmission rates and variable length for high transmission rates).

**Conclusions:**

These findings highlight the importance of obtaining early estimates of pandemic severity and provide guidance to public health decision-makers for effectively tailoring school closures strategies in response to a newly emergent influenza pandemic.

## Background

Influenza pandemics affect millions of people worldwide, exacting significant costs on the global economy in terms of illness, deaths, medical resources, and loss of productivity. In 2009, the influenza A (H1N1) pandemic challenged the world’s socio-economic system and forced public health agencies to take aggressive measures to mitigate its social and economic impact in some parts of the world. It demonstrated the importance of non-pharmaceutical interventions prior to the distribution of an effective vaccine [1]. However, during the early emergence of a new influenza pandemic, estimates of the transmissibility and severity (i.e. case fatality rate) of the new virus are typically based on noisy and sparse data [2]. Public health officials thus face considerable uncertainties when making decisions about early stage pandemic mitigation strategies. School closures offer a radical form of social distancing with great potential to slow the early growth of a pandemic, at significant economic and social costs [3, 4]. Because of this trade-off, knowing the transmissibility and severity of a spreading pandemic are particularly critical to effective school closure policy-making. During the 2009 H1N1 pandemic, schools closures were sporadic in the US, triggered by diverse decision-making entities under variable situations [5, 6] and other countries, e.g. Mexico, implemented nationwide school closures that lasted for about 2 weeks.

In developing school closure policies for future pandemics, researchers and public health officials have used models and quantitative analyses to evaluate their efficacies and costs under possible pandemic scenarios [3, 7]. A number of studies have quantified the impact of school closures on cumulative attack rates, but have not evaluated the social and economic costs of such policies [6, 8–12]. Some of these studies demonstrate school closures can have a significant impact on the basic reproduction number and on the overall spread of disease [4–7, 10, 13, 14]. Other studies have estimated the direct economic cost of influenza and school closures and their indirect impacts on the health care system [3, 4, 15–20]. One advocates, for example, 26 weeks of school closure in conjunction with other policies [4]. For other modes of intervention, a number of studies simultaneously assess epidemiological and economic impacts using stochastic agent-based models of pandemic influenza transmission and decision-analytic approaches [4, 12, 20–22]. While a few of these consider one or very few school closure options among a larger spectrum of intervention measures [4,12,23], none systematically explore the cost effectiveness and epidemiological impact of a large set of school closure policies nor assess the political, social or economic viability of such policies for mild pandemics such as 2009 H1N1 [24].

Here, we combine a decision analytic approach with economic and mathematical infectious disease modeling to estimate the impact of school closures in terms of both epidemiological and cost effectiveness. We consider several scenarios for the transmissibility and severity of the disease, and evaluate diverse closure and reopening strategies covering a range of thresholds in school-aged prevalence (SAP) and closure durations.

## Methods

We evaluate a range of pandemic influenza school closure and reopening strategies, under four different scenarios for the transmissibility and severity of the pandemic. The impacts of school closures on influenza attack rates are estimated using an age-specific mass action model, i.e., all individuals act similarly but separately from each other in a homogenously mixed population [25]. Each school closure policy consists of a prevalence-based closure trigger and either a prevalence-based or fixed duration re-opening trigger. Our cost effectiveness analysis followed the recommendations of the Panel on Cost-Effectiveness in Health and Medicine [26], and considered a detailed model of the societal costs of school closures as outlined by Muenning [27].

### Model

We use an age-structured SEIR (Susceptible-Exposed-Infectious-Recovered) model [28] of pandemic influenza transmission, based on population data for the state of Texas from FedStat [29] (see Table 1). Infected individuals are assumed to enter a latent period (with average duration of three days) during which they are symptom-free, followed by an infectious period (with average duration of six days) during which they are both symptomatic and infectious [12] See Additional file 1 for one-way sensitivity analyses on both latent and infectious periods.

**Table 1:** Model parameters

**Sociological Parameters**								**Reference(s)**
**Age Groups**				**Population**				
*<= 5*				2,019,138				FedStat [29]
*>5 < =18*				4,655,105				
*> = 19*				17,612,731				
*Total*				24,326,974				
**% With Children <16**				53.58				FedStat [29]
**Average Daily Salary ($/day)**				135.25				
**Employment Rate (%)**				93				
**Single Parents (%)**				20				Lempel et al. [3]
**Working parents missing work (%)**				14				
**Days Work Missed**				2.5 (Couples), 5 (Single Parents)				
**Discount Rate (%)**				3				[4,26]
**Cost of Monitoring Infections ($)**				100				Assumed
**Contact Rates (people/day)**								Mossong et al. [30]
**Schools open**		*Adults*			*Children*			
*Adults*		7			1			
*Children*		6			10			
**Schools closed**		*Adults*			*Children*			
*Adults*		7			1			
*Children*		4			2			
**Transmission Rates (β)**		**Adults**			**Children**			Cauchemez et al. [8]
		*Min*	*Most Likely*	*Max*	*Min*	*Most Likely*	*Max*	
**R**_**0**_ **~ [1.1-1.5]**	*Adults*	0.123	0.145	0.168	0.141	0.167	0.193	
	*Children*	0.227	0.269	0.31	0.262	0.309	0.257	
**R**_**0**_ **~ [1.5-2.1]**	*Adults*	0.178	0.201	0.234	0.205	0.231	0.269	
	*Children*	0.33	0.372	0.434	0.38	0.427	0.499	
**Other Influenza Parameters**								
**Latent period (1/μ)**		3 days						Gojovic et al. [12]
**Infectious period (1/γ)**		6 days						
**Case Fatality Rates (CFR)**		*Adults*			*Children*			
*Low Severity Scenario*		0.159%			0.01%			Presanis et al. [31]
*High Severity Scenario*		2%			2%			Chowell et al. [32]

We considered two levels of pandemic severity: relatively low case fatality rates (CFR) of 0.01% for the school age population and 0.16% for the adult population, based on estimates for the 2009 (H1N1) pandemic [31] and higher CFR’s (2% for both of the age groups), as estimated for the 1918 pandemic [32].

On the other hand, estimates for the basic reproduction number (*R*_0_) of 2009 H1N1 pandemic influenza (and other strains of pandemic flu) range between 1.1 and 2.1 [33]. Accordingly, we considered two transmission rate scenarios: *R*_0_ values are sampled from age-specific triangular distributions with values ranging either from 1.1 to 1.5 (low transmission scenario) with mean 1.3 or from 1.5 to 2.1 (high transmission scenario) with mean 1.8 (Table 1). Triangular distributions were chosen because of a lack of consensus about the distribution of reproduction numbers for pandemic influenza; they have only three relatively tangible parameters (minimum, maximum, and most likely value) that are readily subjected to sensitivity analyses across likely ranges.

Mixing rates between age groups in the model were estimated from the contact numbers reported in Mossong et al. [30] (Table 1). For estimates of mixing rates during school closures, contacts taking place in schools were excluded, i.e. there is no compensatory contact included in the model during school closures. Age-specific transmission rates were then calculated based on the mixing rates and *R*_0_ (see Additional file 1 and Table 1).

The mathematical formulation of the age-structured SEIR model is given in Additional file 1. Numerical simulations were initiated with five exposed individuals per 1000 in the school-aged population. For each combination of epidemic scenario (high/low transmission with high/low severity) and school closure policy option, we ran 200 simulations, each based on a random sample of *R*_*0*_, i.e. sampling values from the corresponding probability distributions (Table 1).

### School closure policies and the decision pathway

The possible closure triggers were a range of SAP thresholds (prevalence in children ages 5–18), based on the assumption that prevalence in this age group can be estimated in real-time from school absenteeism data. For the low transmission scenario, the SAP triggers were 0.5%, 0.8%, 1.1%, 1.4%, 1.7% and 2.0%. For the high transmission scenario, the SAP triggers included these options plus 3%, 4%, 5% and 6%. These were constrained by the peak prevalence predicted by the model (2.2% and 7.9% for the school age population with a peak after 113 and 71 days in the low and high transmission scenarios, respectively). The ten reopening policy options were fixed duration closures of 1, 2, 3, 4, 8, 12, and 24 weeks and declines in SAP to 75%, 50% or 25% of that measured at the time of school closure. Policies that reopen schools based on drops in SAP depend on continual epidemiological surveillance, which can be costly and unreliable. Our model assumes that existing surveillance systems allow one-week delayed estimates of SAP, and that additional monitoring would allow real-time estimates of SAP, at a cost. In both cases, we assume that the SAP estimates are exact.

Fixed duration options may be more practical than prevalence-based re-openings, because fixed durations allow public health agencies, schools and families to plan in advance. However, we do not explicitly model any additional inconvenience or costs associated with trigger-based re-openings and the study is limited in its ability to capture the magnitude of how rational individuals will adapt economic behavior to minimize costs.

**Figure 1:**
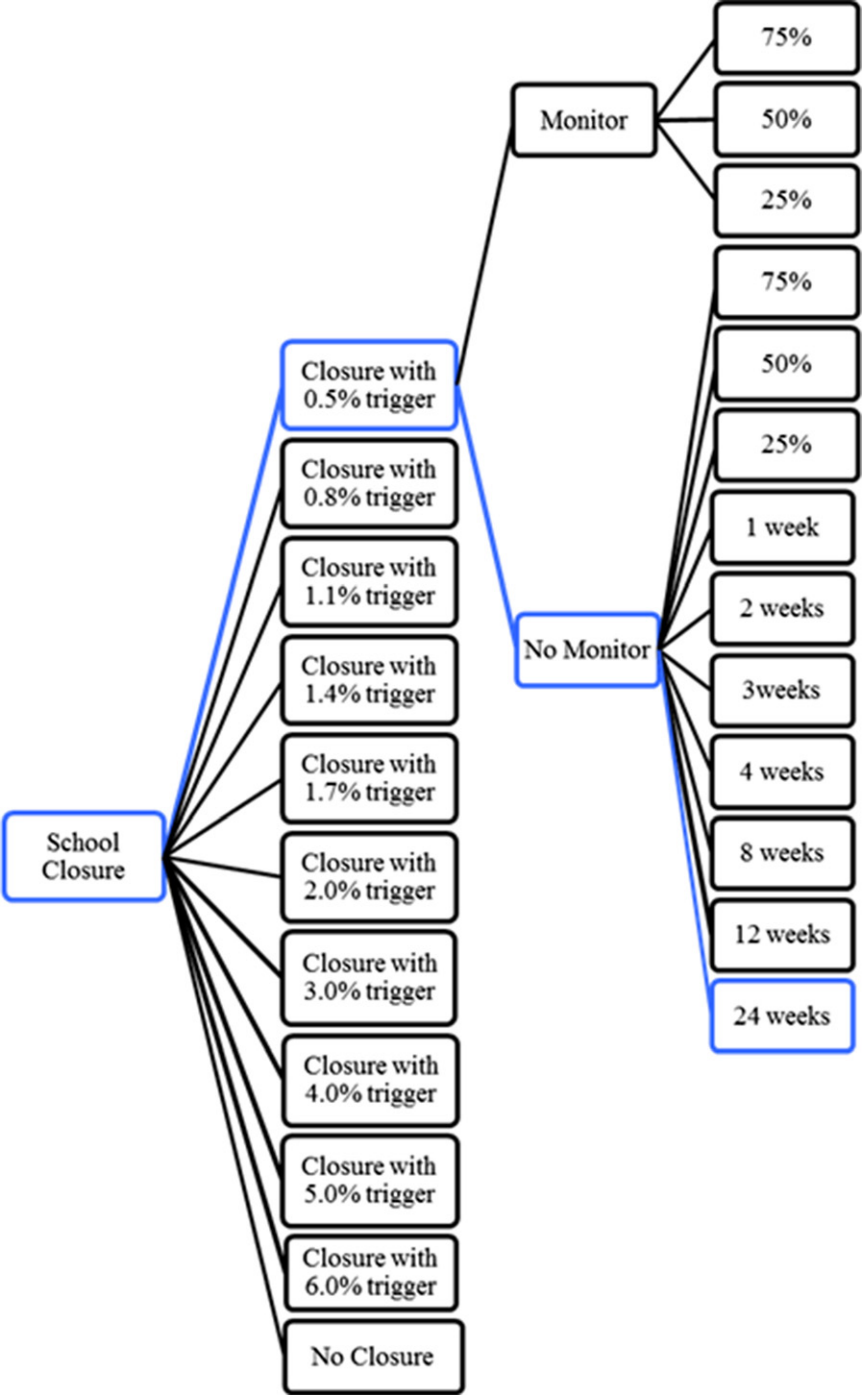
**School closure policy pathway for high transmission scenarios. **The full tree for the 0.5% school-age prevalence closure trigger is shown. All other closure triggers have the same decision options as the 0.5% trigger, but are not depicted. The policy option in blue (24-week closure triggered by 0.5% school-aged prevalence) is one of the efficient options in the tree under both high transmission scenarios (high and low severity).

Figure 1 illustrates a small portion of the policy tree for the high transmission scenarios. Every school closure policy option is represented by a unique path from the left to the right through the tree. For example, the policy following the top-most branches is a school closure that (a) is triggered to close when SAP reaches 0.5%, (b) institutes additional monitoring for changes in SAP during the closure, and (c) is triggered to re-open when the SAP drops to 25% of that observed at the time of the initial closure.

### Cost calculation and cost effectiveness analysis

We estimated the societal costs and health outcomes for each policy and for a base case of no school closure during the first 180 days of the pandemic (prior to the availability of vaccines).

#### School closure costs

Our calculations included costs of work absenteeism during school closure events for parents forced to stay home with their healthy children and did not include the hospitalization costs (see Additional file 1). We assumed 2.5 worker days per week loss for the working co-parents and 5 worker days per week loss for working single parents, consistent with values used in other studies [3, 4]. Based on the 2009 Texas census data, we assumed that 20% of working adults are single parents [29]. Worker productivity loss due to school closure absenteeism was calculated according to the Human Capital Method, as in [3, 4, 34]: average salary plus benefits times the number of days work lost.

#### Health outcomes

For each policy, we determined several health outcomes: number of cases, cases averted by intervention, number of deaths, years of life saved (YLS), total costs, and quality adjusted life-years (QALYs). According to guidelines for program evaluation from the societal perspective [4, 26], we applied a discount rate of 3% per annum (range = 1.5% to 6%) to both health and economic outcomes. QALYs were calculated as one of the health outcomes, based on the total years of life saved and the health related quality of life (HRQL) scores for Influenza-Like-Illnesses (ILI) of different age groups considered in this study [26, 27, 35]. The QALY is a measure of health outcome that assigns each period of time a preference-based weight corresponding to the quality of life during that period, i.e., a weight of one corresponds to perfect health and a weight of zero corresponds to a health state equivalent to death [26].

We calculated incremental cost effectiveness ratios (ICER) of each strategy (i.e., the incremental expense incurred for gaining the incremental health outcome when comparing two alternative strategies) as follows:

(1) $$\text{ICER}=\frac{\text{Total}\;\text{Cost}\;\text{of}\;\text{Strategy}\;\left(B\right)-\text{Total}\;\text{Cost}\;\text{of}\;\text{Strategy}\left(A\right)}{\text{Total}\;\text{QALY}\left(B\right)-\text{Total}\;\text{QALY}\left(A\right)}$$

We report the ICERs of only the “efficient” closure policies, that is, those that have the greatest positive impact for any level of investment. By convention, all other strategies are considered *dominated* and were omitted from the cost-effectiveness ratio calculations. (Results for weakly dominated strategies are provided in Tables A6.1-A6.4 in the Additional file 1). We identified efficient frontiers for each scenario by simultaneously considering the total cost and total QALYs of the interventions.

### Sensitivity analyses

To assess the robustness of our results to uncertainties in critical inputs, we performed deterministic, one-way sensitivity analyses on several critical model parameters: the discounting rate for calculating QALY’s lost due to mortality, fraction of working parents that miss work during a school closure, average daily salary, case fatality rates, percent of working parents that are single, and overall employment rate. We varied each parameter from half of its base value to twice its base value, and assessed the impact on the predicted ICER’s.

## Results

We evaluate the epidemiological and cost effectiveness of a spectrum of school closure policies for mitigating an influenza pandemic in the US under four different pandemic scenarios: low transmission-low severity, low transmission-high severity, high transmission-low severity, and high transmission-high severity. In the low transmission scenarios, our model predicts a base case (no intervention) cumulative attack rate (CAR) of 41% for the school age population and 31% for the adult population. Analyzing 200 simulations with different samples of *R*_*0*_ for each scenario-policy combination, we found that the attack rates tend to decrease with the duration of the closure, and increase with the initial prevalence trigger, that is, with the delay in closing schools (Tables 2 and 3).

**Table 2:** Average Cumulative Attack Rates (CAR) for school age population and adults under the low transmission scenarios (standard deviations in parenthesis)

R0 [1.1-1.5]		**CAR for different durations (weeks) of closure for each triggers (%)**						
**Closure Triggers**	**Populations**	1	2	3	4	8	12	24
0.50%	Students	39.12	35.63	32.02	28.35	14.28	6.15	2.39
		(5.8)	(6.9)	(6.3)	(7.3)	(4.9)	(1.9)	(1.7)
	Adults	42.45	39.30	35.88	32.30	18.52	10.52	6.88
		(4.7)	(5.6)	(5.2)	(6.4)	(4.9)	(2.5)	(2.9)
0.80%	Students	38.34	34.67	30.88	26.93	13.60	6.81	4.02
		(6.2)	(6.7)	(7.1)	(6.2)	(4.2)	(1.4)	(1.2)
	Adults	41.88	38.57	35.01	31.31	18.56	12.15	9.07
		(4.8)	(5.6)	(5.8)	(5.7)	(4.2)	(2.3)	(2.0)
1.10%	Students	37.74	33.46	29.38	25.30	13.38	8.46	6.66
		(6.2)	(6.4)	(6.3)	(5.9)	(2.8)	(1.3)	(1.0)
	Adults	41.49	37.75	34.09	30.36	19.46	15.05	13.38
		(5.1)	(5.3)	(5.5)	(5.2)	(3.2)	(2.8)	(2.2)
1.40%	Students	37.12	32.54	28.01	24.13	14.10	10.62	9.43
		(6.2)	(6.0)	(5.9)	(5.4)	(2.0)	(1.9)	(1.8)
	Adults	41.08	37.18	33.27	29.89	21.06	18.14	16.90
		(5.1)	(4.9)	(5.0)	(4.6)	(2.9)	(3.7)	(3.2)
1.70%	Students	36.69	31.63	27.32	23.63	15.58	13.27	12.40
		(5.6)	(5.6)	(5.2)	(4.5)	(1.9)	(2.8)	(1.8)
	Adults	40.82	36.70	33.09	29.96	23.26	21.45	20.80
		(4.6)	(4.5)	(4.7)	(3.9)	(3.5)	(4.2)	(4.5)
2.00%	Students	36.35	31.17	27.10	24.03	17.97	16.53	16.50
		(6.1)	(4.6)	(3.7)	(2.7)	(3.3)	(4.6)	(4.0)
	Adults	40.69	36.61	33.34	30.85	26.10	25.07	25.05
		(4.8)	(3.8)	(3.5)	(2.8)	(4.6)	(5.5)	(5.9)

**Table 3:** Average Cumulative Attack Rates (CAR) for school age population and adults under the high transmission scenarios (standard deviations in parentheses)

R0 [1.5-2.1]		**CAR for different durations (weeks) of closure for each triggers (%)**						
**Closure Triggers**	**Populations**	1	2	3	4	8	12	24
0.50%	Students	73.96	73.71	73.36	73.04	71.21	65.28	10.10
		(2.7)	(3.3)	(3.3)	(3.5)	(3.7)	(3.1)	(1.4)
	Adults	76.90	76.76	76.62	76.46	75.36	71.14	35.51
		(2.2)	(2.2)	(2.1)	(1.9)	(2.1)	(3.8)	(6.0)
0.80%	Students	73.80	73.28	72.88	72.50	69.80	61.16	11.40
		(3.5)	(3.7)	(3.9)	(3.6)	(4.6)	(8.1)	(1.0)
	Adults	76.78	76.62	76.44	76.24	74.67	68.71	38.14
		(2.1)	(10)	(2.0)	(2.1)	(2.6)	(4.9)	(3.4)
1.10%	Students	73.58	72.95	72.37	71.85	68.31	56.65	12.90
		(3.8)	(3.3)	(3.6)	(3.2)	(4.8)	(7.9)	(1.0)
	Adults	76.74	76.47	76.21	75.94	73.88	66.16	40.34
		(2.2)	(2.1)	(2.2)	(2.3)	(2.6)	(4.4)	(3.4)
1.40%	Students	73.32	72.51	71.90	71.13	66.54	51.80	14.55
		(3.1)	(3.2)	(3.1)	3.8)	(4.3)	(7.8)	(9.0)
	Adults	76.61	76.30	75.97	75.66	73.02	63.52	42.44
		(2.0)	(3.2)	(2.1)	(2.2)	(2.2)	(5.3)	(2.2)
1.70%	Students	73.22	72.15	71.28	70.47	64.81	47.38	15.90
		(3.5)	(2.0)	(3.5)	(4.2)	(5.8)	(9.5)	(1.0)
	Adults	76.58	76.13	75.74	75.35	72.13	61.26	44.02
		(2.2)	(3.5)	(3.5)	(2.2)	(3.1)	(5.4)	(4.2)
2.00%	Students	73.00	71.80	70.74	69.62	62.98	43.48	17.30
		(3.4)	(2.2)	(2.0)	(3.9)	(5.7)	(9.4)	(1.5)
	Adults	76.52	75.96	75.51	74.99	71.22	59.25	45.15
		(2.3)	(3.7)	(3.9)	(1.8)	(3.2)	(5.1)	(2.5)
3%	Students	72.28	70.49	68.75	67.21	56.47	31.82	21.80
		(3.4)	(1.9)	(2.0)	(3.9)	(7.1)	(4.8)	(2.0)
	Adults	53.33	52.80	52.24	51.73	47.59	39.04	49.40
		(2.1)	(4.2)	(3.2)	(2.1)	(3.9)	(3.4)	(2.9)
4%	Students	71.56	69.04	66.80	64.47	50.04	32.20	26.60
		(3.4)	(3.5)	(2.1)	(4.2)	(7.6)	(2.7)	(2.0)
	Adults	53.12	52.39	51.70	50.91	45.47	38.92	52.95
		(2.2)	(2.2)	(4.0)	(2.3)	(4.0)	(3.3)	(4.4)
5%	Students	70.87	67.63	64.68	61.61	45.40	33.34	30.40
		(3.5)	(3.8)	(2.1)	(4.7)	(5.1)	(1.9)	(3.5)
	Adults	52.94	51.97	51.04	50.03	44.13	39.98	55.90
		(2.2)	(2.2)	(4.5)	(2.5)	(3.2)	(3.4)	(4.0)
6%	Students	70.16	66.24	62.58	58.91	43.41	36.35	35.30
		(3.8)	(3.7)	(4.1)	(5.0)	(3.1)	(3.0)	(4.0)
	Adults	52.74	51.57	50.43	49.21	43.86	41.45	58.99
		(2.3)	(2.3)	(2.7)	(3.1)	(3.1)	(3.3)	(4.0)

**Figure 2:**
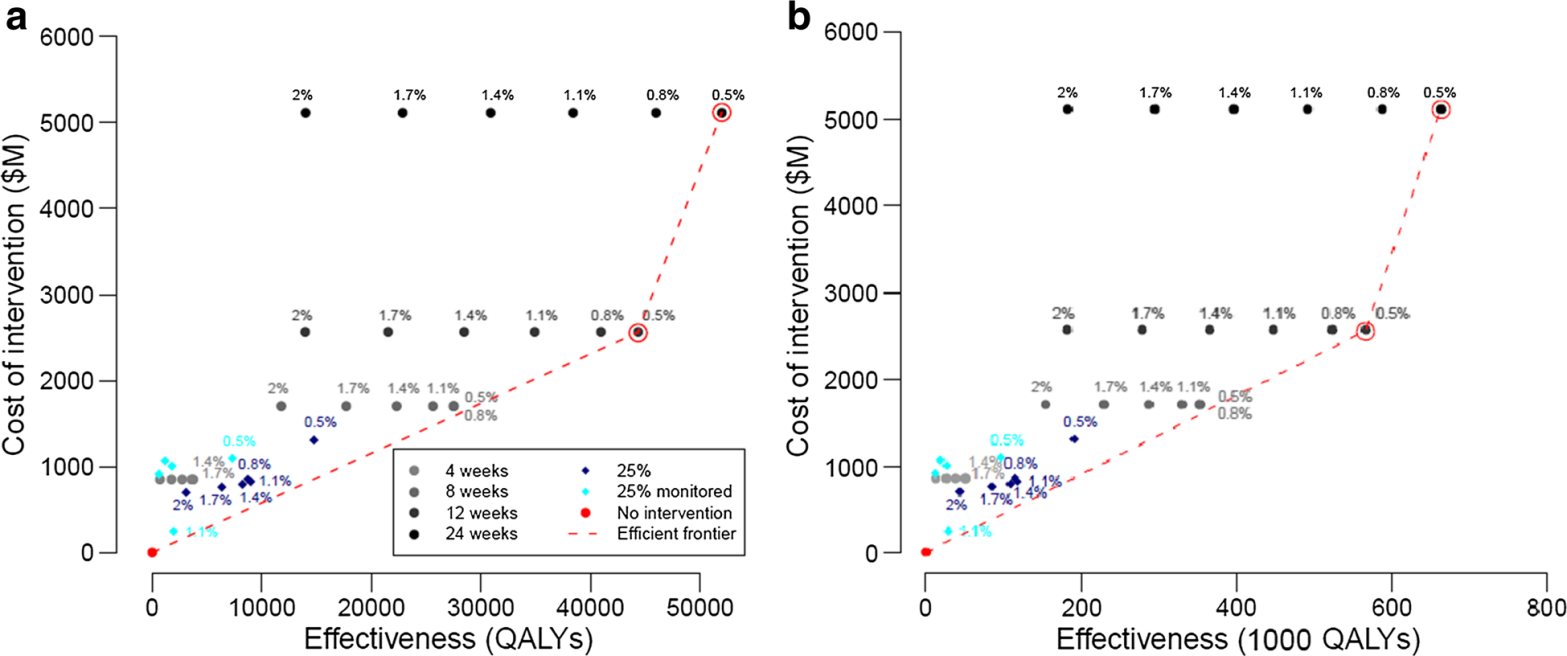
**a: Cost and effectiveness comparison of school closure strategies with different closure triggers for low transmission-low CFR scenario. Red circles indicate efficient strategies. b: **Cost and effectiveness comparison of school closure strategies with different closure triggers for low transmission-high CFR scenario. Red circles indicate efficient strategies.

For the low transmission-low severity and low transmission-high severity scenarios, only the lowest trigger value yields efficient strategies: a 0.5% SAP closure trigger followed by either a 12-week closure ($2.56 billion societal cost and total of 44,300 QALYs) or a 24-week closure ($5.12 billion societal cost and 51,900 QALYs) (Figure 2a and b). For the 12-week closure policy, as the severity increases from a case fatality rate of less than 0.2% to 2%, the incremental cost effectiveness ratio (ICER), that is, the cost per QALY gain, decreases from $57,700 to $4,500 and the expected number of deaths averted rises from 4,250 to 84,188 (however, the proportion of deaths averted increases more modestly from 0.67 to 0.73). For a 24-week closure policy, as the severity increases, the ICER decreases from $334,800 to $26,400 and the expected number of deaths averted increase from 4,981 to 96,702 (the proportion of deaths averted increases from 0.79 to 0.84) (Tables 4 and 5).

**Table 4:** Incremental Cost Effectiveness Ratio (ICER) of the effective closure strategies under the low transmission-low severity scenario

**Intervention**	**Total Deaths**	**YOL Saved**	**Total QALYs Gained**	**Total Closure Cost ($)**	**ICER ($/QALY)**
No closure	6,340	-		0.00	-
0.5%,12w	2,090	133,612.88	44,300	2,560,372,219	57,700
0.5%,24w	1,359	156,209.94	51,900	5,120,744,439	334,800

**Table 5:** Incremental Cost Effectiveness Ratio (ICER) of the effective closure strategies under the low transmission-high severity scenario

**Intervention**	**Total Deaths**	**YOL Saved**	**Total QALYs Gained**	**Total Closure Cost ($)**	**ICER ($/QALY)**
No closure	115,780	-	-	0.00	-
0.5%,12w	31,592	3,748,812.28	566,500	2,560,372,219	4,500
0.5%,24w	19,078	4,256,198.65	663,600	5,120,744,439	26,400

**Figure 3:**
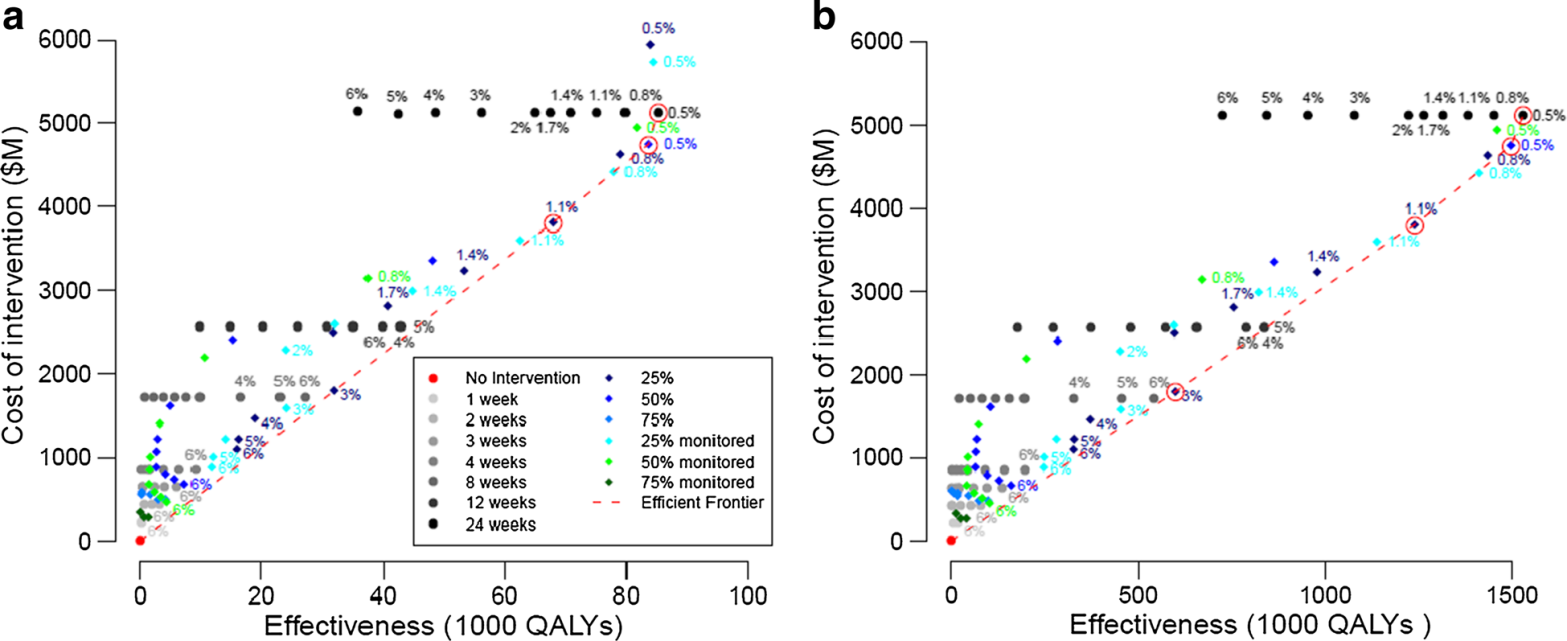
**a: Cost and effectiveness comparison of school closure strategies with different closure triggers for high transmission-low CFR scenario. Red circles indicate efficient strategies. b: **Cost and effectiveness comparison of school closure strategies with different closure triggers for high transmission-high CFR scenario. Red circles indicate efficient strategies.

**Figure 4:**
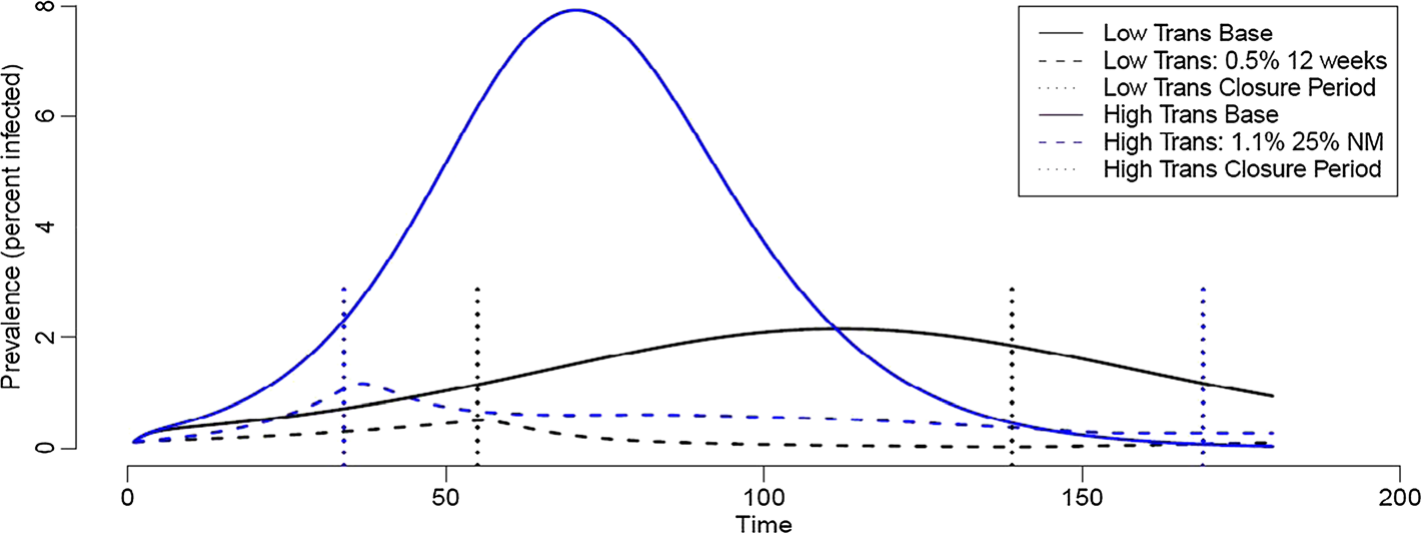
**Total influenza prevalence curves with and without school closures under low transmission (black) and high transmission (blue) scenarios. **Dashed lines show a typical epidemic curve under a cost effective closure policy (based on one simulation). Vertical dotted lines indicate beginning and end of each school closure.

For the high transmission scenarios, our model predicts that 72% of the school age population and 52% of the adult population will become infected. Based on an evaluation of total costs and total QALYs for each school closure strategy, three efficient strategies emerged for the high transmission-low severity scenario (Figure 3a): (i) a 1.1% closure trigger coupled with a non-monitored prevalence-based reopening trigger (specifically, a decrease in SAP to 25% of the original value, that is, a decrease in SAP from 1.1% to 0.275%), (ii) a 0.5% closure trigger with non-monitored prevalence-based reopening trigger (50% decrease in the SAP from the closure trigger), and (iii) a 0.5% closure trigger followed by 24 weeks of closure. Under the first of these policies, the model predicts that schools will close for an average of 125 days (standard deviation 3.67 days) while prevalence declines to the re-opening threshold (Figure 4). Thus, the closure duration falls between the fixed closure options of 12 and 24 weeks. A non-monitored (NM) policy assumes that accurate estimates of SAP are made throughout the closure period with a one-week lag. For the high transmission-high severity, two additional efficient strategies exist; these are 3% closure trigger with non-monitored prevalence-based reopening until SAP drops to 25% of the trigger and 0.8% closure trigger with monitoring (M) based reopening until SAP drops to 25% of the original trigger value (See Figure 3b).

As in the low transmission scenarios, the efficient policies save more lives and have considerably lower ICERs under the high severity scenario than under the low severity scenario (Tables 6 and 7). For example, for the 1.1% SAP trigger coupled with reopening after a reduction in SAP to 25% of its original value (1.1%, NM25%), the incremental cost per QALY gained decreases from $56,000 to $3,100 and the number of deaths averted with school closure increases from 4,081 to 187,307 (the proportion of deaths averted increases from 0.27 to 0.42).

**Table 6:** Incremental Cost Effectiveness Ratio (ICER) of the effective closure strategies under the high transmission-low severity scenario

**Intervention**	**Total Deaths**	**YOL Saved**	**Total QALYs Gained**	**Total Closure Cost ($)**	**ICER ($/QALY)**
No closure	15,182	-	0.00	0.00	-
1.1%,NM25%	11,101	129,858.17	67,900	3,810,077,708	56,100
0.5%,NM50%	8,667	204,696.57	83,600	4,754,976,979	60,200
0.5%,24w	7,167	251,156.96	85,200	5,120,744,439	223,800

**Table 7:** Incremental Cost Effectiveness Ratio (ICER) of the effective closure strategies under the high transmission-high severity scenario

**Intervention**	**Total Deaths**	**YOL Saved**	**Total QALYs Gained**	**Total Closure Cost ($)**	**ICER ($/QALY)**
No closure	444,157	-	0	0.00	-
3%,NM25%	255,762	6,577,339.87	599,300	1,798,356,678	3,000
1.1%,NM25%	256,850	6,524,695.19	1,239,800	3,810,077,708	3,100
0.8%,M25%	255,517	6,587,498.52	1,411,800	4,419,690,141	3,500
0.5%,NM50%	250,337	6,660,573.77	1,497,300	4,754,976,979	3,900
0.5%,24w	249,206	6,889,044.94	1,529,600	5,120,744,439	11,300

Our analyses suggest that efficient strategies depend on the transmission rate of the strain (Tables 3, 4, 5, 6, 7). Although there is some overlap in the efficient sets for the low and high transmission rate scenarios, there are also notable differences. For a relatively slowly spreading strain, an early implementation and relatively long closure (0.5%, 12w or 0.5%, 24w) is efficient. For more rapidly spreading strains, later closures (prevalence thresholds ranging from 0.5% to 3%) are viable, but require variable durations. Specifically, the 3%,NM 25%; 1.1%,NM25%; 0.8%,M25%; and 0.5%,NM50% strategies are predicted to last 59, 125, 145, and 155 days, respectively (with standard deviations of 2.50, 3.28, 3.50, and 4.12 respectively). The ICERs of the efficient strategies are generally predicted to be lower with higher transmissibility and higher severity (Tables 4, 5, 6, 7).

The World Health Organization (WHO) suggests that health interventions be designated *cost-effective* if they deliver QALYs at a cost less than three times a nation’s per capita GDP and *very cost-effective* if the cost per QALY is less than the country’s per capita GDP [36]. To assess the cost effectiveness of school closure policies, we considered closure durations relative to the US per capita GDP in 2009 (approximately $46,000 according to [36]). Our analyses show that for the low transmission-low severity scenario, a 0.5% prevalence closure trigger followed by a 12-week closure ($57,700 per QALY gain) is the only effective and cost effective strategy (i.e., its ICER is less than three times the US per capita GDP). For the low transmission-high severity scenario, a 0.5% prevalence closure trigger followed by either a 12-week closure ($4,500 per QALY gain) or 24-week closure ($26,300 per QALY gain) meets the very cost effective threshold. Thus, the 5%, 24w would be the preferred policy, as it is the largest one (conferring the greatest number of QALYs) with a cost-effectiveness ratio below the willingness-to-pay threshold.

For the high transmission-low severity scenario, a 1.1% closure trigger coupled with a non-monitored prevalence-based reopening trigger (a decrease in SAP to 0.275%), and a 0.5% closure trigger with non-monitored prevalence-based reopening trigger (50% decrease in the SAP to 0.25%) are cost effective but not very cost effective, with costs of $56,000 per QALY gain and $60,200 per QALY gain, respectively. The latter of the two is larger (higher total QALY’s gained), and thus would be the preferred choice. Finally, for the high transmission-high severity scenario, all five efficient strategies meet the very cost effective threshold, with the 0.5% SAP trigger, 24-week closure being the preferred program. Our cost estimates are comparable to published values (Table 8). For the low transmission-low severity scenario, influenza is predicted to cost the State of Texas 0.023–0.5% of its GDP.

**Table 8:** Comparison to published analyses

	**Cost of Influenza**	**Cost of Closure**	**Closure Duration**	**Population**	**$ Amount**
Sadique et al.	-	0.2-1% GDP	12 weeks	England	0.2-1.2 Billion Sterlin
Lempel et al.	-	0.1-0.3% GDP	4 weeks	US	10-47 Billion $
Smith et al.	3.3-4.3% GDP	-	4 weeks	UK	85.8 -97.6Billion Sterling
Brouwers et al.	2-3% GDP	-	-	Sweden	2.5 Billion SEK
Sander et al.	-	-	26 weeks	US	2720 $/person
Araz et al.		0.023-0.5% GDP	1-24 weeks	Texas	0.2-5.12 Billion $

**Figure 5:**
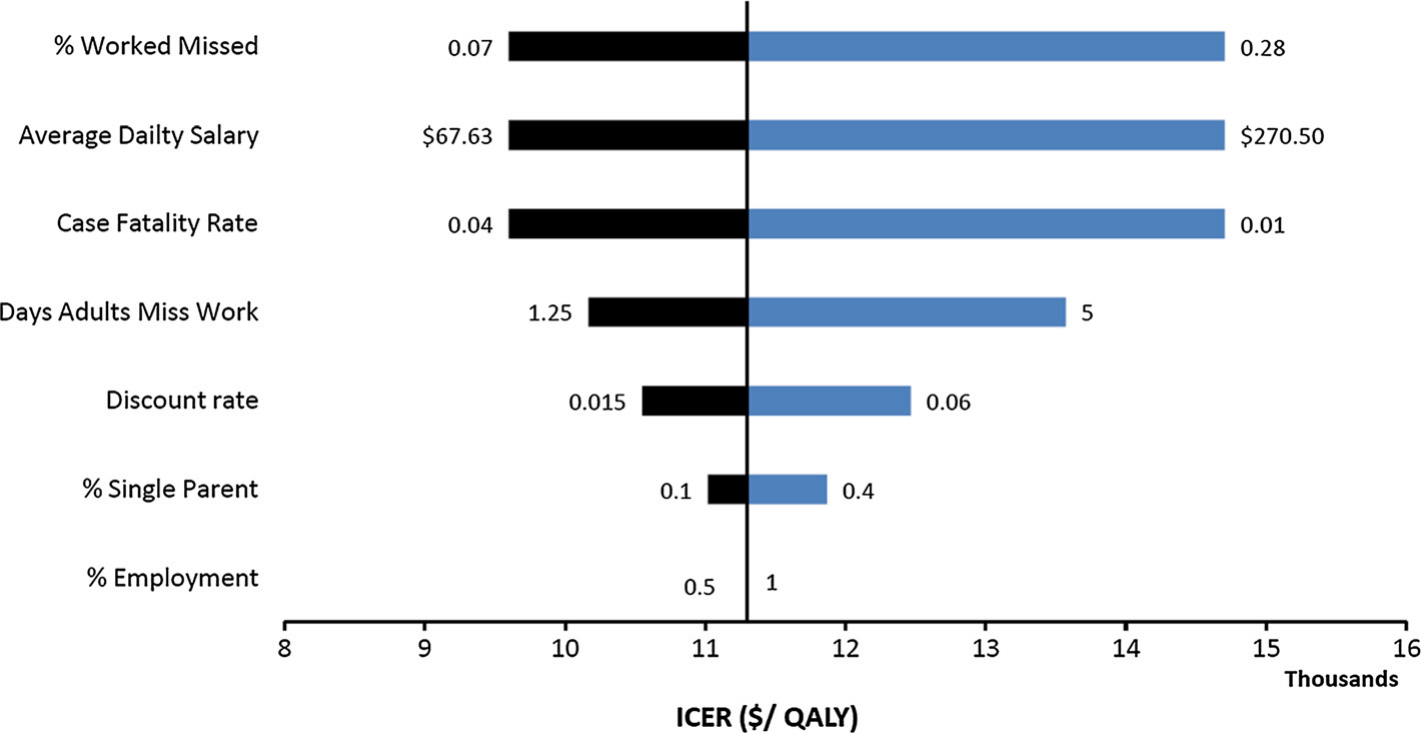
**Tornado diagram comparing the relative impact of input variables on the ICER for the preferred closure policy (0.5% SAP trigger, 24-week) under the High transmission-High severity scenario. ** The width of the bars indicates the uncertainty associated with each parameter as it ranges from 50% of its base value to two times of its base value, as given in Additional file 1.

For both high and low transmission pandemics, the predicted ICER values varied considerably with the percentage of working adults who will miss work due to influenza, average daily salary, and case fatality rate (CFR). For example, we illustrate this with a tornado diagram for the preferred strategy under the high transmission-high severity pandemic scenario (Figure 5). Increases in any of the parameters, except the CFR, lead to increases in the ICER of the school closure policies. Conversely, decreases in pandemic severity (CFR) lead to increases in ICER of the closure policy. None of these parameter perturbations cause the policy to lose its *very cost effective* designation, that is, the ICER remains well below the willingness-to-pay threshold of $46,000. The parameters influence the ICER values similarly for the cost effective policies under the other three pandemic influenza scenarios (Figures A4.1-A4.3 in Additional file 1). While none of the perturbations cause the preferred policies rise above the cost effectiveness willingness-to-pay threshold, some extreme parameter perturbations are predicted to move policies from very cost effective to cost effective (by increasing the ICER) or, vice versa, from cost effective to very cost effective (by reducing the ICER).

### Discussion and conclusions

School closure interventions, in general social distancing interventions and population behavior changes, will lead to a delayed impact of the overall pandemic by reducing the peak of the current pandemic wave and overall epidemic size. However, it is expected that the temporarily spared susceptible population during the first wave can be affected during subsequent second or third pandemic waves. It is also important to note that it may take number of weeks before reliable estimates of pandemic severity could be inferred and this could impact the effectiveness of policy decisions on whether school closures should be triggered. This was the case during the 2009 A/H1N1 pandemic in Mexico; when the Mexican government decided to close schools across the country based on the available information, there was no reliable estimate of the case fatality ratio.

According to our model, school closures can significantly reduce the total number of influenza cases, but the epidemiological impact and societal costs of a school closure critically depend on the timing and duration of the closure. In the early days of a pandemic, there is typically considerable uncertainty about its transmission rate (or *R*_0_) and its severity (or CFR). Policy makers face two interrelated decisions: (1) whether or not to implement a school closure policy; and (2) if so, which one. The answer to the first question depends on the predicted effectiveness and cost effectiveness of the effective policy options. We find that relatively few closure policies are efficient under the low transmissibility scenarios, while several are efficient under the high transmissibility scenarios. The answer to the second question depends on both the transmission rate and severity of the pandemic. Slower spreading pandemics call for early triggers and relatively long duration closures (12 weeks and 24 weeks), regardless of severity; and more rapidly spreading pandemics allow for higher triggers (e.g. 1.1% and 3%) coupled with moderate durations (averages of 125 days and 59 days, respectively), depending on the severity of the strain (see Additional file 1 for timing of the triggers for both cases). Although we assumed that the SAP estimates are exact in the model and single step errors, e.g. a policy intending to close school at a SAP of 0.8% actually closing school at 0.5% or 1.1%, do not make large differences in CARs, it is worth mentioning that these kind of errors can generate dramatic switches in the effectiveness of policy implementation, e.g. a switch from a cost effective strategy to a dominated or weakly dominated strategy (e.g. see low transmission, low CFR scenario in the Additional file 1 Table A6.1).

The policy options included fixed duration closures and re-opening triggers based on decreases in school-aged disease prevalence, both with and without additional surveillance to improve real-time prevalence estimates. Our model assumed that, in the absence of monitoring, decision-makers receive accurate estimates of prevalence with a one-week delay, and monitoring simply removes the delay. Although the direct cost of monitoring is assumed to be low, most of the efficient policies are either fixed duration or have non-monitored reopening triggers. This suggests that slightly lower reopening thresholds than those considered may provide a better balance between costs of closure and health outcome. Similarly, the efficiency of several trigger-based re-opening policies may indicate that there are better fixed durations than the values considered.

In summary, we have integrated a mathematical model of influenza transmission dynamics into a cost-effectiveness analytic framework for evaluating a wide range of school closure and reopening policies with respect to their societal costs and health impacts. The presented rigorous approach of this paper can be adapted to evaluate and compare a variety of non-pharmaceutical, vaccine, and antiviral policy options for influenza. We have found that the transmission rate and case fatality rate of a spreading pandemic can dramatically impact whether or not a school closure policy is efficient and cost effective. Although not surprising, this highlights the importance of obtaining early and reliable estimates of pandemic severity to public health decision-making.

## Competing interests

The authors declare that they have no competing interest.

## Authors’ contribution

In this study, OA conducted the majority of the analyses and was the principal author of the manuscript. LM and PD designed the study and directed its implementation, including quality assurance and control, and preparation of the manuscript. AG and DP provided critical input into the analytic techniques and preparation of the manuscript. SB and BG contributed to the implementation of the models and analysis of the model results. All authors read and approved the final manuscript.

## Pre-publication history

The pre-publication history for this paper can be accessed here:

http://www.biomedcentral.com/1471-2458/12/449/prepub

## Supplementary Material

Additional file 1 Appendix 1–6 [37] Click here for file
