# Supplementary material for: Simulating school closure policies for cost effective pandemic decision making
Source: BMC Public Health. 2012 Jun 18;12:449. doi: 10.1186/1471-2458-12-449 (PMC3495022; doi:10.1186/1471-2458-12-449)
Supplement: Additional file 1 — Appendix 1–6 [37] [file 1471-2458-12-449-S1.doc]

**Appendix 1: Sensitivity Analyses on latent period (mu) and infectious period (gamma)**

Figure A1.1: Top panel-Sensitivity analysis on latent period for high transmissibility scenario, ranging from 1 day to 5 days (base case scenario value was 3 days). Bottom Panel-Sensitivity analysis on infectious period for high transmissibility scenario, ranging from 4 day to 8 days (base case scenario value was 6 days).

**Appendix 2: Transmission rate calculations based on a given R_0_**

We use published estimates of *R*_0_ and age-specific contact patterns to calculate age-specific transmission rates. Specifically, we estimate age-specific triangle distributions of transmission rates from which our stochastic simulations sample. Following Keeling and Rohani (2008), we use the formula given in (2).


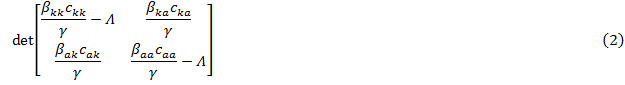


We solve the characteristic polynomial of (3) to obtain the desired transmission rates.


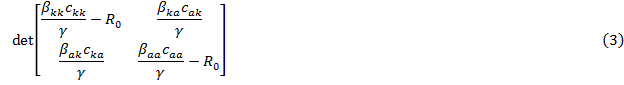


**Appendix 3: Mathematical formulation of the disease transmission models**

**A3.1 Model for Children**


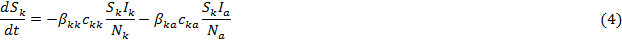


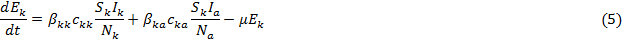


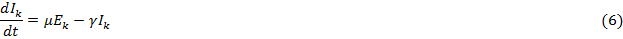


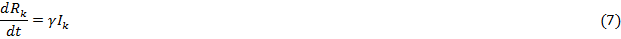


**A3.2 Model for Adults**


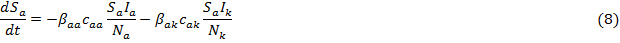


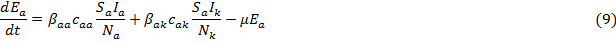


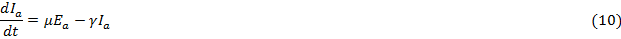


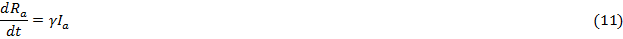


**Appendix 4: Calculating Cost of School Closures**

Cost of School Closure (CC):

- 1. AK : % Adults with kids (< 16)
  2. PH : % parents staying home due to closure
  3. *l*: length of school closure (weeks)
  4. AP : Adult population
  5. *w*: Average daily wage
  6. *ER*: Employment rate
  7. SP: % of Single Parents in the Population
  8. *t_1_*: missed days for non-single parents during a week
  9. *t_2_*: missed days for single parents during a week

CC= AK*(1-SP)* *l* * *t_1_**PH**w**ER+AK*SP* *l* * *t_2_**PH**w**ER (A3.1)

Table A4.a: Relative impact of input parameters on the ICER for the high transmission-high severity scenario.

|  | Corresponding Input Value | | | Output Value | | | Percent |
| --- | --- | --- | --- | --- | --- | --- | --- |
| Input Variable | Low Output | Base Case | High Output | Low | Base | High | Swing^2 |
| % Miss work | 0.07 | 0.14 | 0.28 | 9597.66 | 11300.31 | 14705.62 | 27.7% |
| Average Daily Salary | $67.63 | $135.25 | $270.50 | 9597.66 | 11300.31 | 14705.62 | 27.7% |
| Case Fatality Rate | 0.04 | 0.02 | 0.01 | 9597.66 | 11300.31 | 14705.62 | 27.7% |
| Days Adults Miss Work | 1.25 | 2.5 | 5 | 10165.21 | 11300.31 | 13570.52 | 12.3% |
| Discount rate | 0.015 | 0.03 | 0.06 | 10550.19 | 11300.31 | 12465.48 | 3.9% |
| % Single Parent | 0.1 | 0.2 | 0.4 | 11016.54 | 11300.31 | 11867.86 | 0.8% |
| % Employment | 0.5 | 0.918 | 1 | 11300.31 | 11300.31 | 11300.31 | 0.0% |

Figure A4.1: Tornado diagram comparing the relative impact of input variables on the ICER for the Low transmission-Low severity scenario. The widths of the bars indicate the uncertainty associated with each parameter as it ranges from 50% of its base value to two times of its base value, as given in Table A3b.

Figure A4.2: Tornado diagram comparing the relative impact of input variables on the ICER for the Low transmission-High severity scenario. The widths of the bars indicate the uncertainty associated with each parameter as it ranges from 50% of its base value to two times of its base value.

Figure A4.3: Tornado diagram comparing the relative impact of input variables on the ICER for the High transmission-Low severity scenario. The widths of the bars indicate the uncertainty associated with each parameter as it ranges from 50% of its base value to two times of its base value.

Table A4.b: Relative impact of input parameters on the ICER for the low transmission-low severity scenario.

|  | Corresponding Input Value | | | Output Value | | | Percent |
| --- | --- | --- | --- | --- | --- | --- | --- |
| Input Variable | Low Output | Base Case | High Output | Low | Base | High | Swing^2 |
| % Miss work | 0.07 | 0.14 | 0.28 | 27976.70 | 57708.07 | 117170.80 | 29.6% |
| Average Daily Salary | $67.63 | $135.25 | $270.50 | 27976.70 | 57708.07 | 117170.80 | 29.6% |
| Case Fatality Rate | 0.003 | 0.00159 | 0.0008 | 29802.94 | 57708.07 | 116075.46 | 27.7% |
| Days Adults Miss Work | 1.25 | 2.5 | 5 | 37887.16 | 57708.07 | 97349.89 | 13.1% |
| % Single parent | 0.10 | 0.20 | 0.40 | 57704.40 | 57708.07 | 57715.40 | 0.0% |
| Discount rate | 0.02 | 0.03 | 0.06 | 57708.07 | 57708.07 | 57708.07 | 0.0% |
| % Employment | 0.5 | 0.918 | 1 | 57708.07 | 57708.07 | 57708.07 | 0.0% |

**Appendix 5**

Table A5: Time to triggers for both low and high transmission scenarios

| **Triggers (%)** | **Time to Trigger for Low Transmission Case (days)** | **Time to Trigger for High Transmission Case (days)** |
| --- | --- | --- |
| **0.5** | 1 | 1 |
| **0.8** | 10 | 6 |
| **1.1** | 24 | 10 |
| **1.4** | 35 | 14 |
| **1.7** | 46 | 17 |
| **2** | 57 | 19 |
| **3** | - | 26 |
| **4** | - | 31 |
| **5** | - | 36 |
| **6** | - | 40 |

**Appendix 6**

Table A6.1: ICER calculations of the undominated strategies for low transmission and low CFR scenario including the weakly dominated strategies in the table

| **Intervention** | **Total Deaths** | **YOL Saved** | **Total QALYs Gained** | **Total Closure**  **Cost ($)** | **ICER** |
| --- | --- | --- | --- | --- | --- |
| No closure | 6,340 | - | 0 | 0.00 | - |
| 1.1%,M25% | 6,142 | 8714.23 | 1,900 | 243,844,973 | WD |
| 2%,NM25% | 6,031 | 12477.33 | 3,000 | 701,054,298 | WD |
| 1.7%,NM25% | 5,720 | 22016.43 | 6,300 | 762,015,541 | WD |
| 1.4%,NM25% | 5,539 | 27479.38 | 8,200 | 792,496,163 | WD |
| 1.1%,NM25% | 5,469 | 29550.16 | 8,900 | 822,976,784 | WD |
| 0.5%,NM25% | 4,918 | 46322.66 | 14,700 | 1,310,666,731 | WD |
| 0.5%,8w | 3,696 | 84051.54 | 27,500 | 1,706,914,813 | WD |
| 0.5%,12w | 2,090 | 133612.88 | 44,300 | 2,560,372,219 | 57,700 |
| 0.5%,24w | 1,359 | 156209.94 | 51,900 | 5,120,744,439 | 334,800 |

Table A6.2: ICER calculations of the undominated strategies for low transmission and high CFR scenario including the weakly dominated strategies in the table

| **Intervention** | **Total Deaths** | **YOL Saved** | **Total QALYs Gained** | **Total Closure Cost ($)** | **ICER** |
| --- | --- | --- | --- | --- | --- |
| No closure | 115,780 | - | 0 | 0.00 | - |
| 1.1%,M25% | 99,392 | 1,044,638.73 | 28,431.39 | 243,844,973 | WD |
| 2%,NM25% | 95,865 | 1,235,134.98 | 43,483.42 | 701,054,298 | WD |
| 1.7%,NM25% | 90,934 | 1,423,516.73 | 84,774.05 | 762,015,541 | WD |
| 1.4%,NM25% | 88,359 | 1,513,098.63 | 108,615.58 | 792,496,163 | WD |
| 1.1%,NM25% | 87,759 | 1,519,439.78 | 117,946.94 | 822,976,784 | WD |
| 0.5%,NM25% | 79,495 | 1,820,832.46 | 190,865.41 | 1,310,666,731 | WD |
| 0.5%,8w | 58,790 | 2,654,183.10 | 353,237.68 | 1,706,914,813 | WD |
| 0.5%,12w | 31,592 | 3,748,812.28 | 566,533.57 | 2,560,372,219 | 4,500 |
| 0.5%,24w | 19,078 | 4,256,198.65 | 663,695.61 | 5,120,744,439 | 26,300 |

Table A6.3: ICER calculations of the undominated strategies for high transmission and low CFR scenario including the weakly dominated strategies in the table

| **Intervention** | **Total Deaths** | **YOL**  **Saved** | **Total QALYs Gained** | **Total Closure Cost ($)** | **ICER** |
| --- | --- | --- | --- | --- | --- |
| No closure | 15,182 | - | 0 | 0.00 | - |
| 6%, 1w | 15,159 | 1,039.71 | 218.71 | 213,364,351.60 | WD |
| 6%, M75% | 15,050 | 4,516.60 | 1,349.57 | 274,325,594.90 | WD |
| 6%,2w | 14,872 | 10,213.12 | 3,201.62 | 426,728,703.20 | WD |
| 6%,M50% | 14,759 | 13,815.64 | 4,377.45 | 457,209,324.90 | WD |
| 6%,3w | 14,598 | 18,965.95 | 6,057.31 | 640,093,054.90 | WD |
| 6%,NM50% | 14,481 | 22,675.54 | 7,271.29 | 670,573,676.50 | WD |
| 6%,4w | 14,301 | 28,411.44 | 9,147.95 | 853,457,406.50 | WD |
| 6%,M25% | 14,051 | 36,315.28 | 11,746.40 | 883,938,028.10 | WD |
| 5%,M25% | 14,030 | 36,924.85 | 11,975.89 | 1,005,860,515.00 | WD |
| 6%,NM25% | 13,650 | 49,019.05 | 15,929.96 | 1,097,302,380.00 | WD |
| 1.7%,NM50% | 14,902 | 9,163.66 | 2,900.01 | 1,219,224,866.00 | WD |
| 4%,M25% | 13,831 | 43,046.47 | 14,052.81 | 1,219,224,866.00 | WD |
| 5%,NM25% | 13,622 | 49,791.10 | 16,222.64 | 1,219,224,866.00 | WD |
| 4%,NM25% | 13,361 | 57,836.23 | 18,955.24 | 1,463,069,840.00 | WD |
| 3%,M25% | 12,881 | 72,681.49 | 23,973.11 | 1,584,992,326.00 | WD |
| 6%,8w | 12,573 | 83,093.99 | 27,147.96 | 1,706,914,813.00 | WD |
| 3%,NM25% | 12,115 | 96,742.43 | 31,956.90 | 1,798,356,678.00 | WD |
| 5%,12w | 11,052 | 131,034.47 | 43,000.24 | 2,560,372,219.00 | WD |
| 1.4%,M25% | 10,881 | 135,239.80 | 44,844.18 | 2,987,100,923.00 | WD |
| 1.4%,NM25% | 10,075 | 160,614.13 | 53,241.03 | 3,230,945,896.00 | WD |
| 1.1%,M25% | 9,183 | 188,420.22 | 62,557.92 | 3,596,713,356.00 | WD |
| 1.1%,NM25% | 8,667 | 204,696.57 | 67,932.02 | 3,810,077,708.00 | 56,000 |
| 0.8%,M25% | 7,705 | 234,666.48 | 77,983.65 | 4,419,690,141.00 | WD |
| 0.8%,NM25% | 7,596 | 238,153.33 | 79,117.35 | 4,633,054,492.00 | WD |
| 0.5%,NM50% | 7,167 | 251,156.96 | 83,615.84 | 4,754,976,979.00 | 60,200 |
| 0.5%,24w | 11,101 | 129,858.17 | 85,250.02 | 5,120,744,439.00 | 223,800 |

Table A6.4: ICER calculations of the undominated strategies for high transmission and high CFR scenario, including the weakly dominated strategies in the table

| **Intervention** | **Total Deaths** | **YOL Saved** | **Total QALYs Gained** | **Total Closure Cost ($)** | **ICER** |
| --- | --- | --- | --- | --- | --- |
| No closure | 444,157 | - | 0 | 0.00 | - |
| 6%, 1w | 245,233 | 7,077,301.13 | 17,688.73 | 213,364,352 | WD |
| 6%, M75% | 242,771 | 7,192,610.53 | 40,821.82 | 274,325,595 | WD |
| 6%,2w | 239,239 | 7,358,398.60 | 78,791.91 | 426,728,703 | WD |
| 6%,M50% | 227,677 | 7,892,149.17 | 102,390.86 | 457,209,325 | WD |
| 6%,3w | 232,870 | 7,654,436.18 | 136,232.33 | 640,093,055 | WD |
| 6%,NM50% | 219,408 | 8,268,428.34 | 160,244.09 | 670,573,677 | WD |
| 6%,4w | 197,191 | 9,280,515.91 | 197,411.60 | 853,457,407 | WD |
| 6%,M25% | 243,163 | 7,174,111.27 | 247,500.35 | 883,938,028 | WD |
| 5%,M25% | 245,714 | 7,053,678.06 | 248,740.23 | 1,005,860,515 | WD |
| 6%,NM25% | 251,626 | 6,774,523.00 | 327,367.52 | 1,097,302,380 | WD |
| 5%,NM25% | 252,885 | 6,712,948.98 | 328,755.52 | 1,219,224,866 | WD |
| 4%,NM25% | 254,287 | 6,646,318.12 | 371,832.28 | 1,463,069,840 | WD |
| 3%,M25% | 251,759 | 6,765,366.27 | 453,524.37 | 1,584,992,326 | WD |
| 6%,8w | 171,478 | 10,465,341.38 | 541,902.67 | 1,706,914,813 | WD |
| 3%,NM25% | 255,762 | 6,577,339.87 | 599,371.26 | 1,798,356,678 | 3,000 |
| 5%,12w | 165,831 | 10,733,578.49 | 837,467.26 | 2,560,372,219 | WD |
| 1.4%,NM25% | 256,285 | 6,550,614.93 | 978,372.76 | 3,230,945,896 | WD |
| 1.1%,M25% | 254,506 | 6,633,902.36 | 1,138,769.69 | 3,596,713,356 | WD |
| 1.1%,NM25% | 256,850 | 6,524,695.19 | 1,239,832.94 | 3,810,077,708 | 3,100 |
| 0.8%,M25% | 255,517 | 6,587,498.52 | 1,411,836.03 | 4,419,690,141 | 3,500 |
| 0.8%,NM25% | 257,138 | 6,508,828.85 | 1,435,071.81 | 4,633,054,492 | WD |
| 0.5%,NM50% | 98,337 | 13,460,573.77 | 1,497,331.85 | 4,754,976,979 | 3,900 |
| 0.5%,24w | 249,206 | 6,889,044.94 | 1,529,624.92 | 5,120,744,439 | 11,300 |
